# Magnesium Intake, *C*-Reactive Protein, and Muscle Mass in Adolescents

**DOI:** 10.3390/nu14142882

**Published:** 2022-07-14

**Authors:** Yutong Dong, Li Chen, Bernard Gutin, Ying Huang, Yanbin Dong, Haidong Zhu

**Affiliations:** 1 Georgia Prevention Institute, Department of Medicine, Medical College of Georgia, Augusta University, Augusta, GA 30912, USA; ytdong24@gmail.com (Y.D.); lichen1@augusta.edu (L.C.); bernardgutin@yahoo.com (B.G.); yihuang@augusta.edu (Y.H.); ydong@augusta.edu (Y.D.); 2 Internal Medicine Residency Program, Department of Medicine, New York University Langone Health, New York, NY 10016, USA

**Keywords:** magnesium, inflammation, muscle mass, adolescents

## Abstract

Background: Adult studies have suggested that magnesium intake may regulate *C*-reactive protein (CRP) and muscle mass, known risk factors for cardiometabolic diseases. Given the large deficiencies in magnesium intake in adolescents, we aimed to investigate sex and race differences in dietary magnesium intake and test the hypothesis that lower magnesium intake is associated with higher CRP and lower muscle mass. Methods: A total of 766 black and white adolescents, 14 to 18 years old (51% black; 50% female) were previously recruited. Diet was assessed with four to seven independent 24-h recalls. Body composition was measured by dual-energy X-ray absorptiometry. High-sensitivity CRP (hs-CRP), leptin, resistin, and adiponectin were measured using fasting blood samples by ELISA. Results: There were sex and race differences in the daily consumption of magnesium. The average daily magnesium intakes were 200.66 ± 7.09 mg and 205.03 ± 7.05 mg for males and females, respectively, far below the recommended amounts of 410 mg for males and 360 mg for females. White subjects (217.95 ± 6.81 mg/day) consumed more than black subjects (187.75 ± 6.92 mg/day). Almost none of the adolescents met the recommendations. Adjusted multiple linear regressions revealed that lower magnesium intake was associated with higher hs-CRP and lower fat-free mass (FFM) (*p*-values < 0.05). Higher hs-CRP was associated with lower FFM. Moreover, an interaction between magnesium intake and hs-CRP on FFM was identified (*p*-value < 0.05). Lower magnesium intake amplified the inverse relationships between hs-CRP and FFM (*p*-values < 0.05). Conclusion: Magnesium consumption in our adolescents was far below daily recommended levels with male and black subjects consuming less than female and white subjects. Lower magnesium intake was associated with higher CRP and lower muscle mass. Low magnesium intake may also augment the inverse relationship between CRP and FFM.

## 1. Introduction

Magnesium (Mg) is an essential dietary element for humans involved in key biological processes. Hypomagnesemia is associated with muscle and bone metabolism conditions including sarcopenia and osteoporosis, neuromuscular conduction disorders, arrythmias including Torsade de pointes, and clinical hypocalcemia. Magnesium is known to be involved in various enzymatic, membrane, calcium antagonistic and structural functions. Magnesium is absorbed mainly from the intestinal tract via passive and active transport, where 99% would eventually be stored intracellularly. Active magnesium transport occurs through the combined actions of transient receptor potential melastatin-6 and 7 (TRPM6/7), which are apical membrane magnesium entry channels. A majority of the magnesium ion is ultimately filtered with some reabsorption in the renal tubules. A mutation in TRPM6 has been shown to lead to decreased absorption and increased renal wasting. Magnesium is suggested to be regulated by parathyroid hormone and the renin–angiotensin–aldosterone system (RAAS). It also serves as a secondary messenger for insulin signaling. Low serum magnesium is found in type 2 diabetic patients and is associated with insulin resistance [1]. Chronically low magnesium levels can induce low grade inflammation via imbalance of calcium homeostasis, oxidative stress from mitochondrial apoptosis and cytokine activation, myocardium electrical remodeling, chronic activation of RAAS, a well-established proinflammatory system and insulin resistance via signaling disruption [2].

Although low magnesium intake is an important risk factor for various diseases including cardiometabolic [3,4,5], the majority of American adults and adolescents consume inadequate dietary magnesium, a common trait of the Western diet [6,7]. In fact, approximately 60% of the US population consumed inadequate magnesium, with the greatest deficiencies occurring between ages 14 to 18 years old. The 2007–2010 National Health and Nutrition Examination Survey (NHANES) identified that 75% of males and 87% of females consumed less than their estimated average requirements in this age group [8]. 

Adult population studies have reported inverse associations of magnesium intake and cardiometabolic risks, including high inflammation and low muscle mass [9,10]. Furthermore, inflammation and muscle mass may be closely related, and more studies are needed to investigate their relationships [11,12,13]. A study on NHANES 2001–2010 in 14,338 adults found that lower magnesium intake (from food and supplements) was associated with increased cardiometabolic risks, including elevated *C*-reactive protein (CRP) levels. CRP is a key inflammatory molecule that can adversely affect cardiometabolic health [14,15,16,17,18]. 

A meta-analysis of magnesium intake in 24,473 adults suggested that magnesium may regulate our health through muscle activity [19]. Low muscle mass is also associated with increased cardiometabolic risks and mortality [20,21,22]. A study in adult women suggested that magnesium intake may attenuate the inverse relationship between CRP and muscle mass [9]. These results are supported by experimental animal studies, in which magnesium deficiency increased inflammation and reduced protein synthesis [23,24,25]. Despite our current knowledge, tremendous gaps remain concerning the relationships between magnesium intake and health risks in both adults and adolescents, particularly their underlying mechanisms [7]. Additionally, adolescent sex and race differences in dietary magnesium consumption in adolescents have yet to be investigated.

Data on magnesium intake and inflammation in adolescents is scarce. Few studies have reported the relationships among dietary magnesium intake, CRP, and muscle mass together in the youth population. This study had two major aims: (1) to determine the sex and race differences in dietary magnesium intake among adolescents; and (2) to test the hypothesis that lower magnesium intake is associated with a higher level of CRP and lower level of muscle mass.

## 2. Materials and Methods

### 2.1. Participants

A cross-sectional study was conducted in a cohort of apparently healthy black and white adolescents aged 14 to 18 years old (n = 766: 51% black; 50% females) [26]. Participants were recruited, from local public high schools in the Augusta, Georgia area (located in southeast US), to take part in the Lifestyle, Adiposity, and Cardiovascular Health in Youth (LACHY) study. Demographic information obtained from the school systems was used to select schools that enrolled both black and white students. After receiving approval from the county superintendents and school principals, flyers were distributed to all students in the selected schools. Subjects were asked to self-identify their ethnicity. Subjects who identified themselves as being white/Caucasian or black/African American were eligible for the study. There are relatively few youths from other racial/ethnic groups in the Augusta area, and they were excluded from the study. Interested students who responded to the flyers and called the institute to participate were screened over the telephone to determine preliminary eligibility. Participants were excluded with the following self-reported conditions: taking current medications or diagnosed with chronic medical conditions that could affect growth, maturation, physical activity (PA), nutritional status, or metabolism. Pregnant females were also excluded from the study. Adolescents who passed the telephone screening were invited to the Georgia Prevention Institute, accompanied by their parents if they were minors, to learn about the study.

### 2.2. Ethics Statement

The Institutional Review Board at the Medical College of Georgia, Augusta University approved this project (Augusta, GA, USA, protocol #622505). Flyers, approved by school superintendents and principals, were distributed to selected high schools. Written informed consent was obtained from all adult subjects and parents/guardians of those younger than 18 years of age. Each participant was assigned a unique subject number. All data were anonymized and de-identified prior to analysis.

### 2.3. Anthropometry and Body Composition

Demographic data, including age, sex, and race, were collected through questionnaire. Height and weight were obtained according to standard procedures, using a wall-mounted stadiometer (Tanita Corporation of American, Arlington Heights, IL, USA) and calibrated electronic scale (model CN2OL; Cardinal Detecto, Webb City, MO, USA). Prior to testing each week, the electronic scale was checked for accuracy using known weights. Body mass index (BMI) was calculated as weight (kg) divided by height (m^2^) [27].

Fat-free mass (FFM) mass and fat mass were assessed by dual-energy X-ray absorptiometry (QDR-4500W; Hologic Inc., Waltham, MA, USA). Anthropomorphic phantoms were scanned daily for quality assurance. For determination of measurement reproducibility, a one-way random-effects model, and single-measure intraclass correlation coefficients were calculated in participants 14–18 years of age (n = 219). Each participant was scanned twice within a 7 d period for FFM and fat mass (both r ≥ 0.97) [28].

### 2.4. Biochemical Measurements and Sexual Development

Fasting blood samples were collected from participants and processed within one hour of blood collection. EDTA-plasma tubes were centrifuged at 3000 rpm × 10 min at 9 °C. The tiger top serum tubes were left to sit at room temperature for 30, then centrifuged at 3000 rpm × 10 min at 9 °C. Aliquots were stored at −80 °C until analysis. Plasma biomarkers were measured using high-sensitivity enzyme-linked immunosorbent assay (ELISA). ELISA is a technique to detect the presence of antigens in biological samples. An ELISA, like other types of immunoassays, relies on antibodies to detect a target antigen using highly specific antibody–antigen interactions. In an ELISA assay, the antigen is immobilized to a solid surface. This is done either directly or via the use of a capture antibody itself immobilized on the surface. The antigen is then complexed to a detection antibody conjugated with a molecule amenable for detection such as an enzyme or a fluorophore. Plasma high-sensitivity CRP (hs-CRP) concentrations were assayed using high-sensitivity ELISA (ALPCO Diagnostics) and run in duplicate, with intra- and interassay coefficients of variation (CVs) of 10% and 10.2%, respectively. Adiponectin and resistin were measured in plasma that was assayed in duplicate by ELISA (Linco Research). Intra- and interassay CVs were 7.4% and 8.4%, respectively, for plasma adiponectin and 3.2% and 7.1%, respectively, for plasma resistin. Serum leptin concentrations were assayed using ELISA (R & D Systems) and run in duplicate, with intra- and inter-assay CVs of 2.2% and 5.3%, respectively.

Sexual development of the participants was measured by a five-stage scale, ranging from 1 (prepubertal) to 5 (fully mature) as described by Tanner [29]. Using a sex-specific questionnaire, the subjects reported their sexual maturation stage by comparing their own physical development to the five stages in standard sets of diagrams. A parent or research coordinator then reviewed the results with the youth to make sure they understood the questionnaire. When an individual reported discordant stages of pubic hair and breast or genital development, the higher of the two stages was used.

### 2.5. Physical Activity

The total daily minutes spent for moderate and vigorous PA were assessed using MTI Actigraph monitors (model 7164; MTI Health Services, Fort Walton Beach, FL, USA), uniaxial accelerometers that measure vertical acceleration and deceleration. With epoch length set at 1 min and expressed as counts per minute, the accelerometers started recording when the subject left our institute after the first testing day. The subjects were instructed to (1) wear the monitor for a period of 7 days, (2) remove it for sleep, bathing, and any activity that may cause harm to either the monitor or another person (e.g., during contact sports), and (3) bring the monitor back to us after 1 week. Data from days 1 and 7 were discarded because a full day of information was not available. Movement counts were converted to average minutes per day spent in moderate (3–6 metabolic equivalents) and vigorous (>6 metabolic equivalents) PA by the software accompanying the device.

### 2.6. Dietary Assessment

Dietary intake was assessed with individual, non-consecutive, 24 h recalls that covered the period from midnight to midnight for the previous day. The first two recalls were conducted in person at our institute within 1 week of testing with the use of food models, portion booklets, or serving containers to assist in estimating serving size, and the remaining interviews were conducted by telephone weekly, with all 7 recalls completed within a period of 12 weeks. We sought to obtain 7 recalls from each participant, one for each day of the week. The dietary recalls were conducted by a trained dietitian or dietetic intern. The dietary recalls used the Nutritional Data System for Research (NDS-R 2006) (Nutrition Coordinating Center, University of Minnesota, Minneapolis, MN, USA), which utilizes a multiple pass, computer-assisted interview approach to quantify the food and nutrient intake of individuals, analyze the nutrient composition of menus, and calculate the nutrient composition of food recipes. To minimize subject fatigue with the recall process, which might negatively impact reliability, interviewers were trained in conducting the recalls until these could be routinely completed in 30 min or less. Subjects were not interviewed if they had been ill on the recall day or when the recall day fell on a major holiday. To minimize the potential for under-eating during the time frame for 24 h recalls, subjects were blinded to the telephone recall schedule [28]. Adolescents (95%) provided at least four recalls. Fifty-two percent of the adolescents had recalls for all 7 days. 

### 2.7. Statistical Analysis

Descriptive statistics for variables are presented as mean ± standard deviation. Prior to analysis, homogeneity of variances for all variables was checked using Levene’s test for equality of variances. Race and sex differences were checked for the characteristics assessed, using one-way ANOVA. Pearson Chi-squared test was used to analyze for significant associations between sex and race. Significance was determined when *p*-values were less than 0.05. 

Multivariate regression base models, adjusted for age, sex, race, energy intake (EI), Tanner stage, and BMI, were used to estimate the associations between magnesium intake as the main predictor with hs-CRP and FFM as the outcomes. hs-CRP was natural-log transformed for regression models. Models were then further adjusted for PA to analyze the same associations. Additionally, multivariate regression models, adjusted for the same confounders as above, were used to estimate the association between hs-CRP and FFM. Significance was determined when *p*-values were less than 0.05. 

Moderation base models, adjusted for age, sex, race, EI, Tanner stage, and BMI, were used to analyze whether magnesium intake moderated the association between hs-CRP, log-transformed, and FFM. Models were then further adjusted for PA. Moderation models were estimated by using the PROCESS macro for SPSS. [30] The program displays figures showing conditional slopes at different levels of the moderator (“Low” = 1 standard deviation below the mean, “Medium” = mean, and “High” = 1 standard deviation above). Significance was determined when *p*-values were less than 0.05. 

All statistical analyses were performed via SPSS –IBM Software (version 24.0 SPSS Inc., Chicago, IL, USA) with the significance level set at α = 0.05.

## 3. Results

### 3.1. General Characteristics

The characteristics of our participants (n = 766) are presented in Table 1, and the distributions by sex and race are approximately balanced. Adolescent males (n = 381, 50%) exercised longer, had more FFM, lower fat mass, and lower levels of leptin, resistin, adiponectin than females (all *p*-values < 0.001). Black subjects (n = 389, 51%) had higher BMI, Tanner Stage, more FFM, fat mass, higher leptin, and lower adiponectin than white subjects (all *p*-values < 0.001). 

### 3.2. Magnesium Intake

The average caloric intake of our adolescent males and females fell within the recommended amounts by sex and age group as suggested by the latest Dietary Guidelines for Americans (2015–2020) [31]. Males were found to consume more calories but less dietary magnesium than females (*p*-values < 0.001); black subjects consumed fewer calories and dietary magnesium than white subjects (*p*-value < 0.001). The national recommended daily magnesium intake levels for our age group were 410 mg and 360 mg for males and females, respectively [32]. Our male and female adolescents on average consumed 200.66 ± 7.09 mg and 205.03 ± 7.05 mg of magnesium, respectively. Only 3.1% (n = 12) adolescent males and 3.1% females (n = 12) consumed at their recommended levels. 

### 3.3. Associations of Magnesium Intake with Cardiometabolic Risks

In Table 2, multiple linear regression models adjusted for age, sex, race, EI, Tanner Stage, and BMI revealed that a 1.0 mg decrease in magnesium intake was associated with an increase of 0.3% hs-CRP and a decrease of 0.01 kg FFM (all *p*-values < 0.05). These models, after further adjusting for PA, revealed similar results. No significant sex or race interaction was identified. 

### 3.4. Moderation of Magnesium Intake on C-Reactive Protein and Fat-Free Mass

**Figure 1:**
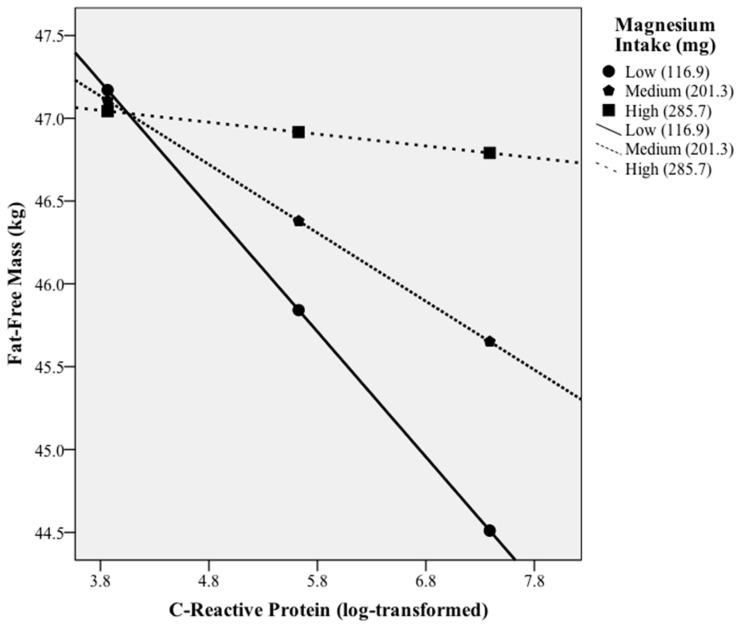
Moderation of high-sensitivity *C*-reactive protein and fat-free soft tissue by magnesium intake. Low = 1 standard deviation below the mean of magnesium intake. Medium = mean of magnesium intake. High = 1 standard deviation above the mean of magnesium intake. N = 543.

hs-CRP was inversely associated with FFM with adjustment of energy intake, age, sex, race, physical activity, Tanner stage, BMI, and PA (*p*-values < 0.05). Interaction between magnesium intake and hs-CRP on FFM was identified (*p*-value < 0.05). Magnesium intake was found to moderate the associations between hs-CRP and FFM (Figure 1). Low magnesium intake (116.9 mg) amplified the inverse association between hs-CRP and FFM (*p*-values < 0.05). The combination of low magnesium intake and high hs-CRP was associated with lowest FFM (*p*-values < 0.05). 

## 4. Discussion

Magnesium is a key regulator of our cardiometabolic health; however, studies in youth are extremely limited. To the best of our knowledge, this is the first adolescent study to report both sex and race intake differences in dietary magnesium intake and to explore the relationships between low magnesium intake, CRP, and muscle mass in healthy adolescents. Our study shows that lower magnesium intake was associated with higher hs-CRP and lower muscle mass. In addition, lower magnesium intake augmented the inverse relationship between hs-CRP and muscle mass.

Our adolescent males and females on average per day consumed 201 mg and 205 mg dietary magnesium, respectively. Our black and white adolescents consumed on average 188 mg and 218 mg per day, respectively. Almost all (96.9%) of our males and females consumed far below the recommended sex-specific magnesium intake for their age group, which are 410 mg for males and 360 mg per day for females [32]. Few studies have investigated magnesium intake in youth. Between 2005 and 2006, NHANES estimated that, within the age group 14 to 18 year-olds, 69% males and 80% females consumed below the average magnesium requirements for their age and sex [6]. In that survey, males consumed on average 300 mg daily and females consumed 211 mg. Comparing to NHANES, the lower magnesium intake of our adolescents may highlight the greater health and diet deficiencies experienced within the Southeast Stroke Belt region. Additionally, our results provided further evidence that youths, between 14 to 18 years old, consumed tremendously low amounts of dietary magnesium regardless of sex and race. Dietary patterns with increased consumption of animal protein, refined sugar and oils, and processed grains negatively influenced the consumption of magnesium [33]. 

We investigated and observed inverse associations of magnesium intake with CRP. A 20-year longitudinal study of 4497 American adults concluded that lower magnesium intake was associated with greater systemic inflammation [34]. A meta-analysis of 32,918 adults concluded that low magnesium intake was associated with high serum CRP levels [10]. Another meta-analysis of RCTs indicated that magnesium supplementation reduced CRP levels among individuals with inflammation (CRP levels > 3 mg/dL) [35]. A most recent meta-analysis also showed that magnesium supplementation significantly decreased CRP [36]. A RCT among diabetic patients showed that magnesium supplementation significantly decreased IL-6 levels, which is another key inflammatory marker [37]. Furthermore, the molecular basis for inflammatory responses may be linked to the modulation of intracellular calcium concentration, where magnesium acts as a calcium antagonist [23,38]. Animal and in vitro studies indicate that magnesium deficiency increases cellular Ca^2+^, which is the signal that results in the priming of cells to give the inflammatory response [39]. Magnesium intake and its effects on CRP require further investigations.

We also observed that magnesium intake was positively associated with muscle mass. Muscle mass may be a key regulator of cardiometabolic risks [12,40,41]. Several cross-sectional studies in adults reported positive associations between magnesium intake and muscle mass and power [9,42,43]. However, a meta-analysis of magnesium supplementation on muscle fitness indicated no significant improvements regarding isokinetic peak torque extension, muscle strength, or muscle power [44]. This relationship is under-investigated in adolescents. One study in seventeen youth athletes observed that low magnesium intake was associated with low muscle mass [45]. The relationship between magnesium intake and muscle mass is particularly important in adolescents because their growth of muscle and soft tissue demand greater magnesium concentration, which further highlights the public health implications of low magnesium intake [46]. Mechanistically, magnesium is suggested to improve muscle mass via reducing serum parathyroid hormone (PTH). Magnesium competes with calcium for binding on calcium-sensing receptors, leading to a decrease in serum PTH [23,47]. Chronic high serum PTH is associated with muscle loss by adversely disrupting muscular energy metabolism, leading to diminished amino acid production and protein synthesis [25,48]. 

Finally, our study reported that dietary magnesium intake may moderate the relationship between CRP and muscle mass. Lower magnesium intake might compound the inverse relationship between CRP and muscle mass. Similar results were found from a previous study investigating magnesium intake, CRP, and muscle mass in adult women [9]. However, the relationships involving magnesium, inflammation, and muscle mass remain unclear and understudied in all populations. Magnesium deficiency can induce inflammatory responses, activating macrophages and promoting an influx of calcium ions intracellularly [10,24]. Through a cascading chain of events, magnesium deficiency can activate the release of proinflammatory cytokines, including interleukin-6 and tumor necrosis factor-alpha, which upregulate CRP [19]. Magnesium deficiency may also upregulate PTH, which is associated with muscle loss [49]. Chronic inflammation may also reduce the regeneration abilities of muscle cells, leading to muscle wasting [50]. A meta-analysis of magnesium supplementation trials in adults concluded that magnesium intake might inhibit inflammation [19]. Moreover, athletes, who had high percentage of FFM, were reported to have low levels of CRP [51]. Our results provide additional evidence for the complex relationships observed among magnesium intake, inflammation, and muscle mass in adolescents. 

Additional underlying mechanisms whereby magnesium intake regulates inflammation and muscle mass may be through insulin signaling pathways. Maintaining serum Mg^2+^ concentrations within the reference range is essential for normal insulin secretion and activity, as well as for the optimal functioning of many enzymes of glucose and energy metabolism. Low serum magnesium is associated with insulin resistance [1]. Mg+2 can regulate insulin secretion from pancreatic beta cells [52]. Mg+2 also regulates peripheral insulin sensitivity through two main signaling pathways: the Ras/mitogen-activated protein kinases pathway (Ras/MAPK), which regulates gene expression and insulin-associated mitogenic effects, and the phosphatidylinositol-3-kinase (PI3K)/Akt (protein kinase B) pathway, responsible for most its metabolic actions. The PI3K/Akt kinase pathway plays a central role in insulin signaling, since its activation leads to phosphorylation of an important number of substrates with key functions in a wide variety of biological processes, including glucose transport stimulation, glycogen and protein synthesis, and lipogenesis. Akt appears to play an important role in insulin metabolic actions, including muscle and adipose tissue glucose uptake through glucose transporter type 4 (GLUT4) translocation from intracellular compartments to the cell membrane. Additionally, Akt participates in the regulation of glycogen synthesis by glycogen synthase kinase 3 inhibition [52]. Moreover, insulin directly suppresses pro-inflammatory transcription factors in immune cells and the subsequent inflammatory mediators through non-metabolic pathways, thus ameliorating inflammation [53]. 

This was an observational study to estimate the average daily magnesium intake in healthy white and black youth age 14–18 years. Almost all (96.9%) of our males and females consumed only half the recommended sex-specific magnesium intake for their age group. Moreover, pregnant youth were excluded from the study. Thus, the risk of magnesium overdose is low in our study. Although rare, some youth may take abnormal dose of magnesium supplements. Magnesium overdose may lead to decreased blood pressure, cardiac arrest, irregular heartbeat, muscle weakness and seizure [54].

There were several notable strengths in our study. First, we reported sex and race differences in magnesium intakes and investigated possible relationships among magnesium intake, inflammation, and muscle mass in adolescents for the first time. Second, we recruited a relatively large and apparently healthy adolescent population with near equal distributions of males and females and of black and white subjects. Third, the repeated collection of four to seven independent 24 h dietary recalls over a 12-week period provided relatively accurate dietary assessments of usual magnesium intake, by reducing bias due to measurement error and random error owing to within-person variability over time [55]. Our study is limited by its cross-sectional nature; thus the associations did not prove causality. Additional limitations including dietary magnesium intake were estimated by 24 h recall method. A single 24 h recall is not considered to be representative of habitual diet at an individual level [56]. Four repeat 24 h recalls were recommended as the most appropriate method of dietary assessment [57]. In our study, we sought to obtained seven 24 h dietary recalls from each participant, one for each day of the week over a 12-week period to better represent the habitual diet. We also tried to minimize errors by averaging seven 24 h dietary recalls. Moreover, the 24 h recalls were conducted by a trained dietitian or dietetic intern. A total of 95% of the adolescents in our study completed 4-day 24 h dietary recalls, and 52% of the adolescents had recalls for all 7 days.

## 5. Conclusions

Our study shows that (1) the vast majority of our healthy adolescents do not consume sufficient magnesium; (2) there are sex and race differences in magnesium intake; and (3) lower dietary magnesium intake is associated with higher CRP and lower muscle mass, independent of energy intake, body weight, and PA. (4) Finally, low magnesium intake may amplify the inverse relationship between CRP and muscle mass. Chronic low intake of dietary magnesium, a common trait of the Western diet, may contribute to high inflammation and low muscle mass. Since CRP and muscle mass play important roles in the pathogenesis of cardiometabolic diseases, increasing dietary magnesium intake may have therapeutic benefits and may be a valuable component of a cardioprotective strategy in adolescents. 

## Figures and Tables

**Table 1:** General characteristics by sex and race.

Characteristics	Demographics				*p*-Value	
	Male	Female	Black	White	Sex	Race
General Demographics						
Number of subjects	381	385	389	377		
Age (years)	16.12 ± 1.25	16.13 ± 1.21	16.18 ± 1.23	16.07 ± 1.20	0.87	0.30
Male/Female Ratio	N/A	N/A	0.98	0.99	N/A	0.95
Black/White Ratio	0.96	0.97	N/A	N/A	0.95	N/A
BMI (kg/m^2^)	22.88 ± 4.74	23.21 ± 3.51	24.32 ± 5.86	22.03 ± 4.09	0.14	<0.001
Physical Activity (minutes)	53.98 ± 31.98	34.06 ± 21.70	44.04 ± 30.38	43.83 ± 27.64	<0.001	0.61
Tanner Stage	4.30 ± 0.77 (n = 371)	4.38 ± 12.32 (n = 375)	4.41 ± 0.73 (n = 368)	4.28 ± 0.73 (n = 378)	0.19	0.02
DEXA Measurements (n)	376	380	372	384		
Fat-Free Mass (kg)	52.75 ± 8.78	40.25 ± 6.03	48.42 ± 10.01	44.59 ± 9.29	<0.001	<0.001
Fat Mass (kg)	13.42 ± 10.06	19.23 ± 10.35	17.35 ± 11.91	15.36 ± 8.46	<0.001	<0.001
Inflammation Measurements						
hs-CRP (mg/L)	0.93 ± 1.79 (n = 307)	1.21 ± 2.48 (n = 329)	1.20 ± 2.4 (n = 295)	0.96 ± 1.94 (n = 341)	0.10	0.17
Leptin (ng/mL)	6.14 ± 8.78 (n = 310)	17.41 ± 13.71 (n = 338)	14.16 ± 14.28 (n = 300)	10.17 ± 11.29 (n = 348)	<0.001	<0.001
Resistin (ng/mL)	10.98 ± 6.09 (n = 324)	12.35 ± 6.34 (n = 339)	11.77 ± 6.26 (n = 314)	11.60 ± 5.12 (n = 349)	<0.001	0.72
Adiponectin (μg/mL)	7.53 ± 4.50 (n = 304)	9.50 ± 5.36 (n = 294)	8.50 ± 5.04 (n = 273)	9.28 ± 5.11 (n = 325)	<0.001	<0.001
Dietary Intake (per day)						
Energy Intake (kcal)	2239.66 ± 627.84	1725.97 ± 570.73	1915.16 ± 651.50	2045.75 ± 647.24	<0.001	<0.001
Magnesium (mg)	200.66 ± 7.09	205.03 ± 7.05	187.75 ± 6.92	217.95 ± 6.81	<0.001	<0.001

Note: N/A = Not Applicable.

**Table 2:** Regressions of magnesium intake on cardiometabolic risks.

Predictors	Magnesium Intake	
	Base Model	Base Model + Physical Activity
hs-CRP	(n = 626) <−0.01 (−0.01, −0.00) *	(n = 542) <−0.01 (−0.01, −0.00) *
Leptin	(n = 638) <−0.01 (−0.01, −0.00) **	(n = 553) <−0.01 (−0.01, −0.00) *
Resistin	(n = 653) <−0.01 (−0.00, 0.00)	(n = 565) <−0.01 (−0.00, 0.00)
Adiponectin	(n = 594) <0.01 (0.00, 0.00)	(n = 513) <0.01 (−0.01, 0.01)
Fat-Free Mass	(n = 745) 0.01 (0.01, 0.02) *	(n = 649) 0.01 (0.01, 0.02) *

Note: Base model was adjusted for energy intake, age, sex, race, physical activity, Tanner stage, and BMI. hs-CRP, leptin, resistin, and adiponectin were log-transformed. * *p*-value < 0.05, ** *p*-value < 0.001.

## Data Availability

The data reported are available upon request.

## Abbreviations

FFM—fat-free mass; CRP—*C*-reactive protein; LACHY—Lifestyle, Adiposity, and Cardiovascular Health in Youth; PA—physical activity; EI—energy intake; BMI—body mass index; PTH—parathyroid hormone.
